# Response to Levetiracetam Treatment and Long-Term Follow-Up in Dogs With Reactive Seizures Due to Probable Exogenous Toxicity

**DOI:** 10.3389/fvets.2021.773942

**Published:** 2021-11-15

**Authors:** Fabio Stabile, Luisa De Risio

**Affiliations:** ^1^Neurology and Neurosurgery Unit, Southfields Veterinary Specialists, Linnaeus, Basildon, United Kingdom; ^2^Linnaeus, Shirley, United Kingdom

**Keywords:** reactive seizures, levetiracetam, intoxication, long-term follow-up, canine epilepsy

## Abstract

Limited information is available on the long-term follow-up and seizure recurrence in dogs with reactive seizures due to suspected exogenous toxicity. The purpose of this study was to report the long-term follow-up of 13 dogs referred to a single referral hospital, diagnosed with reactive seizures and treated with a standardized levetiracetam protocol. All dogs received a loading levetiracetam dose of 60 mg/kg/IV once, followed by a maintenance dose of 20 mg/kg every 8 h as part of an open-label clinical study. Levetiracetam was withdrawn after a 6-months seizure-free period by reducing levetiracetam to 20 mg/kg every 12 h for a 4-week seizure-free period, followed by levetiracetam 20 mg/kg every 24 h for a 4-week seizure-free period, before levetiracetam treatment was stopped. No adverse effects of the treatment were reported. No dogs experienced any seizures after discharge or after levetiracetam withdrawal. Median follow-up time from time of discharge was of 78 months (=6 years 6 months). The result of this study supports the use of levetiracetam for treatment of reactive seizures due to exogenous substance intoxication. Moreover, our results do not support the need for long-term antiepileptic treatment in cases of reactive seizures due to exogenous intoxication.

## Introduction

Reactive seizures (RS) have been defined as seizures “occurring as a natural response from the normal brain to a transient disturbance in function (metabolic or toxic in nature) which is reversible when the cause or disturbance is rectified” (1). Reported prevalence of RS in dogs varies widely among the few studies available in the veterinary literature, ranging from 6.3 to 13.6%. Treatment of RS involves the use of antiseizure drugs (ASD) and treatment of the intrinsic underlying etiology when known (2–6) Levetiracetam (LEV) is a pyrrolidine derivative and is widely used in human medicine for various seizure types. In veterinary medicine, although it is not licensed, it is commonly used due to is safety profile (7), lack of hepatic metabolism (unlike most ASD), and minimal drug interactions. While there is fair evidence for recommending LEV as adjunctive treatment for seizures in dogs, data on efficacy of LEV as monotherapy is scarce (8). Levetiracetam was reported superior to placebo in a randomized, double-masked, placebo-controlled trial for the treatment of cluster seizure and status epilepticus when administered intravenously (IV). This study included 19 dogs (9 receiving LEV and 10 receiving placebo) with epileptic seizure due to various etiologies (9). A prospective open-label clinical trial on intrarectal (IR) LEV administration reported good control of cluster seizures in 21 dogs with idiopathic epilepsy (10). Currently, there is limited information available on long-term follow-up and seizure recurrence in dogs with RS due to suspected exogenous toxicity. The purpose of this study was to report the long-term follow-up of 13 dogs diagnosed with RS treated with LEV, due to highly suspected exogenous toxicity.

## Materials and Methods

This was a prospective open-label clinical study including 13 dogs referred to a single referral hospital (Animal Health Trust, Center for Small Animal Studies) with seizures due to highly suspected exogenous intoxication between January 2009 and December 2015. The study was conducted as a prospective, observational cohort study, approved by the clinical research and ethical committees of the Animal Health Trust (Approval number: AHT42_2009; Approval date: 19th January 2009). Inclusion criteria were: evidence/high suspicion of exogenous toxin ingestion resulting in RS based on anamnestic information given by the owners who witnessed/suspected ingestion of foreign material with or without consecutive presence of gastroinstinal signs (vomiting/hypersalivation/diarrhea), a neurological examination performed by a board-certified neurologist and treatment with LEV (emergency treatment followed by maintenance treatment). Levetiracetam treatment was standardized as follow: at admission all dogs received a LEV loading dose of 60 mg/kg/IV once. The treatment was continued with LEV 20 mg/kg every 8 h orally (PO) or IV until the patient was able to receive medications PO. Levetiracetam treatment was continued in all dogs at a dosage of 20 mg/kg every 8 h (or the nearest dosage based on available formulation and body weight) for a 6-month seizure free period. Levetiracetam was withdrawn by reducing LEV to 20 mg/kg every 12 h PO for a 4-week seizure free period, followed by LEV 20 mg/kg every 24 h PO for a 4-week seizure free period, after which LEV treatment was interrupted. All owners were contacted, as last follow-up, in December 2019. Dogs with a previous history of seizures due to any etiologies prior to the known or suspected intoxication, were excluded from the study. Cluster seizures was defined as two or more seizures in 24 h with complete recovery of consciousness between seizures. Status epilepticus was defined as a single seizure lasting longer than 5 min, or two or more seizures without complete recovery of consciousness in between (1). The following information was collected for each case: signalment (breed, sex, age at presentation, and body weight), seizure type (e.g., single seizure, cluster seizures or status epilepticus) and occurrence, diagnostic investigation results (hematology, serum biochemistry, bile acids/ammonia serum levels, urinalysis, magnetic resonance imaging, and cerebrospinal fluid analysis), LEV adverse effects.

## Results

### Population

Thirteen dogs met the inclusion criteria. The signalment of the 13 dogs is described in Table 1. All dogs presented for cluster seizures of generalized tonic-clonic seizures. Details of seizure activity are included in Table 1. Mean number of seizures from onset to first administration of LEV was 5.38 (range 2–9 RS). The owners of all 13 dogs reported onset of RS after highly suspected ingestion of an unknown toxic substance, although the named substance could not be traced. All dogs were referred after a mean period of 16 h (range 4–24 h) from the first RS. Nine of 13 dogs received subcutaneous apomorphine administration before referral (0.1 mg/kg; EMEDOG^Ⓡ^1 mg/ml, solution for injection for dogs, TVM Animal Health Ltd) to induce emesis. Hematology and serum biochemistry results did not reveal any abnormalities that would result in seizure activity in any of the 13 dogs. In all but 2 dogs (case 5, 6) a bile acid stimulation test and ammonia serum level were performed, and results revealed no significant abnormalities. Urinalysis revealed no significant abnormalities in the 5 dogs in which it was performed (case 1, 2, 8, 9, and 12). Magnetic resonance imaging of the brain and cerebrospinal fluid analysis were performed in all dogs and no abnormalities were detected. Based on the medical history, clinical presentation, clinical course, diagnostic investigation results and follow-up, all dogs were diagnosed with RS triggered by highly suspected ingestion of an unknown toxic substance.

**Table 1:** Signalment, body weight, treatment, and follow-up details of the 13 dogs with highly suspected exogenous toxicity.

	**Patients' details**	**Age at RS onset**	**Pre-referral ApoM treatment**	**Pre-referral emergency ASD treatment**	**Seizure occurrence from onset to discharge**	**Duration of hospitalization**	**Follow-up duration**
1	Labrador, MN, 20 kg	11 m	0.1 mg/kg/SC	Diazepam: 0.5 mg/kg/IR twice	4 seizures in 4 h before referral, 0 seizure after LEV loading	2 days	97 m
2	Cocker Spaniel, MN, 15 kg	24 m	None	None	3 seizures in 5 h before referral, 0 seizures after LEV loading	2 day	96 m
3	Border Collie, FE, 15 kg	6 m	0.1 mg/kg/SC	Diazepam: 1 mg/kg/IR twice	6 seizures in 8 h before referral, 0 seizures after LEV loading.	3 days	117 m
4	Cocker Spaniel, FS, 12 kg	38 m	0.1 mg/kg/SC	Diazepam: 0.5 mg/kg/IR twice	4 seizures in 12 h before referral, 0 seizures after LEV loading.	2 days	Euthanised after 77 m
5	Labrador, MN, 30 kg	60 m	0.1 mg/kg/SC	Diazepam: 1 mg/kg/IR twice	10 seizures in 24 h, of which 9 seizures before referral, 1 seizure after LEV loading.	3 days	Euthanised after 48 m
6	Beagle, MN, 15 kg	48 m	0.1 mg/kg/SC	None	4 seizures in 24 h before referral, 0 seizure after LEV loading.	2 days	60 m
7	French Bull Dog, FS, 11 kg	12 m	0.1 mg/kg/SC	Diazepam: 0.8 mg/kg/IR once	8 seizures in 23 h before referral, 0 seizure after LEV loading.	3 days	Euthanised after 84 m
8	French Bull Dog, FS, 10 kg	18 m	0.1 mg/kg/SC	Diazepam: 1 mg/kg/IR twice	5 seizures in 20 h before referral, 0 after LEV loading.	1 day	62 m
9	Irish Setter, MN, 28 kg	24 m	None	Diazepam: 0.5 mg/kg/IR three times	10 seizures in 24 h, of which 8 seizures before referral, 2 after LEV loading	3 days	Euthanised after 49 m
10	Maltese, FS, 5 kg	14 m	None	Diazepam: 0.5 mg/kg/IR three times	8 seizures in 22 h, of which 7 seizures before referral, 1 after LEV loading.	2 days	108 m
11	German Shorthair Pointer, FS, 30 kg	36 m	None	Diazepam: 0.5 mg/kg/IR once	4 seizures in 12 h before referral, 0 after LEV loading.	1 day	72 m
12	Hungarian Vizsla, FS, 20 kg	36 m	0.1 mg/kg/SC	Diazepam: 0.5 mg/kg/IR four times	3 seizures in 12 h before referral, 0 after LEV loading.	3 days	84 m
13	Weimaraner, FS, 28 kg	24 m	0.1 mg/kg/SC	Diazepam: 0.5 mg/kg/IR three times	6 seizures in 18 h before referral, 0 after LEV loading.	3 days	60 m

*ME, male entire; MN, male neutered; FE, female entire; FS, female spayed; kg, kilograms; m, month; h, hours; CS, cluster seizures; ApoM, apomorphine; ASD, antiseizure drugs; LEV, levetiracetam; SC, subcutaneous; IR, intrarectally; IV, intravenously; PO, orally*.

### Antiseizure Treatment

Details of ASD treatment in each dog are presented in Table 1. Eleven of the 13 dogs received diazepam before referral at a median dose of 0.6 mg/kg/IR (range 0.5–1 mg/kg IR). Two dogs received no treatment prior to referral. According to protocol at admission all dogs received a LEV loading dose of 60 mg/kg/IV once. The treatment was continued with LEV 20 mg/kg every 8 h IV for 24 h in 4/13 dogs until able to receive medications PO (dog 5, 7, 9, and 10), and with LEV 20 mg/kg every 8 h PO in the remaining 9/13 dogs. After initial administration of LEV, the mean number of seizures was 0.38 (range 0–2 RS). Ten dogs experienced no further seizure after the LEV loading administration. Two dogs (dog 5, 10) experienced 1 seizure and 1 dog (dog 9) experienced 2 seizures after the loading LEV administration and no further seizure after the first maintenance (20 mg/kg/IV) LEV administration. In all dogs the LEV withdrawal protocol was followed by all clients without need to interrupt treatment earlier. No adverse effects due to LEV were reported in any of the 13 cases.

### Follow-Up

No dogs experienced any seizures after discharge, nor after LEV withdrawal. Median follow-up time from time of discharge was of 78 months (=6 years 6 months; range: 48–117 months). Nine of the 13 dogs were alive at the time of last follow-up. Dog 4 was euthanised 77 months after discharge, due to recurrence of paraplegia secondary to intervertebral disc herniation. Dog 5 was euthanised after 48 months from discharge because of haemoabdomen due to a ruptured splenic neoplasia and suspected secondary lung metastasis. Dog 7 was euthanised after 84 months from discharge after diagnosis of immune-mediated anemia and dog 9 was euthanised after 49 months from discharge due to gastric torsion.

## Discussion

Relatively little data is available on the efficacy of LEV monotherapy for the treatment of seizures or epilepsy in dogs, and to the best of the authors' knowledge there is no published data on LEV monotherapy for the treatment of RS due to suspected exogenous toxicity in dogs (7, 8). The role of LEV in the maintenance treatment of dogs with idiopathic and structural epilepsy has been described in a few studies (11–13). In this study, LEV was chosen to treat RS due to highly suspected exogenous intoxication due to its favorable metabolic profile compared to other ASD (7). All dogs were treated with a standardized LEV protocol following acute onset of cluster seizures. No seizures occurred after the LEV loading dose in 10 dogs and the first maintenance dose in 3 dogs (dogs 5, 9, and 10). It cannot be ruled out that in these three dogs the pre-referral emergency diazepam administration might have interfered with the plasma concentration of LEV (14). This suggests that LEV was efficacious as ASD in these dogs, however, this is challenging to demonstrate as there was no placebo control group. Similarly, a shorter maintenance treatment duration may have been associated with similar outcomes, but this was not assessed in this study. The decision to continue the LEV for 6 months following the initial presentations was based on current guidelines on initiation of ASD treatment in dogs with idiopathic epilepsy, which include the presence of 2 or more epileptic seizures within a 6-month period and the presence of status epilepticus and cluster seizures, as well as other criteria (15). In fact in the 13 dogs reported in this study the choice of ASD could also be considered further complicated by the knowledge that the signalment of the dogs included in the study could be suggestive of idiopathic epilepsy. Considering that ASD treatment is frequently a life-long commitment, and that dogs affected with RS due to suspected exogenous intoxication might not be needing life-long ASD treatment, our study supports the possibility that LEV monotherapy offers a sensible alternative to the use of other ASD in these cases. In the 13 dogs included in this study, although the exogenous substances could not be traced, the presumptive diagnosis of exogenous intoxications was suspected based on owners' report of sudden onset of cluster seizures after ingestion of unwanted substances and response to antiepileptic treatment. Levetiracetam oral dosage was reduced by ~20% monthly (15). Lack of seizure re-occurrence after LEV discontinuation and a mean follow-up of 78 months (=6 years 6 months; range: 48–117 months) further supports the diagnosis of RS secondary to exogenous toxicity. Interestingly, none of the 13 cases we reported has experienced RS long-term following treatment withdrawal. Similarly, also Zimmerman et al. (4) and Jull et al. (16) reported that no further RS occurred after discharge at a median follow-up time of 2 years 6 months and 2 years, respectively. Our case series therefore strengthen the evidence that cluster seizures triggered by exogenous intoxication does not cause/predispose to recurrent seizures in dogs based on a significantly longer follow-up period (6 years 6 months). Another possible conclusion the reader might take from this study is that the use of LEV might be useful after toxin exposure, equally efficacious or even superior to ASD in cases of reactive seizures due to probable exogenous toxicity. Limitations of the present study are the relatively small sample size which was related to the closure of the institution where the study was conducted, the lack of a control group, and the lack of laboratory evidence of intoxication and named substance, and as such the results of this study should be interpreted with caution. However, these preliminary results can be useful for the practicing veterinarians to inform treatment decision and prognosis discussions with dog owners. In addition, these results provide the foundation for further studies. To the authors' knowledge, this is the first study supporting the use of LEV for treatment of RS due to exogenous substance intoxication. Moreover, our results do not support the need for long-term antiepileptic treatment in cases of RS due to exogenous intoxication.

## Data Availability Statement

The original contributions presented in the study are included in the article/supplementary material, further inquiries can be directed to the corresponding author/s.

## Ethics Statement

The animal study was reviewed and approved by Animal Health Trust Ethical Committee. Written informed consent was obtained from the owners for the participation of their animals in this study.

## Author Contributions

FS and LD have drafted, written, and corrected the paper together. All authors contributed to the article and approved the submitted version.

## Conflict of Interest

The authors declare that the research was conducted in the absence of any commercial or financial relationships that could be construed as a potential conflict of interest.

## Publisher's Note

All claims expressed in this article are solely those of the authors and do not necessarily represent those of their affiliated organizations, or those of the publisher, the editors and the reviewers. Any product that may be evaluated in this article, or claim that may be made by its manufacturer, is not guaranteed or endorsed by the publisher.
